# Using RNA-seq to characterize responses to 4-hydroxyphenylpyruvate dioxygenase (HPPD) inhibitor herbicide resistance in waterhemp (*Amaranthus tuberculatus*)

**DOI:** 10.1186/s12870-019-1795-x

**Published:** 2019-05-06

**Authors:** Daniel R. Kohlhase, Jamie A. O’Rourke, Micheal D. K. Owen, Michelle A. Graham

**Affiliations:** 1 Department of Agronomy, Iowa State University, Ames, IA USA; 2 U.S. Department of Agriculture (USDA)–Agricultural Research Service (ARS), Corn Insects and Crop Genetics Research Unit, Ames, IA USA

**Keywords:** RNA-seq, de novo transcriptome assembly, herbicide resistance, HPPD, *Amaranthus tuberculatus*

## Abstract

**Background:**

Waterhemp (*Amaranthus tuberculatus* (Moq.) J.D. Sauer) is a problem weed commonly found in the Midwestern United States that can cause crippling yield losses for both maize (*Zea mays* L.) and soybean (*Glycine max* L. Merr). In 2011, 4-hydroxyphenylpyruvate-dioxygenase (HPPD, EC 1.13.11.27) inhibitor herbicide resistance was first reported in two waterhemp populations. Since the discovery of HPPD-herbicide resistance, studies have identified the mechanism of resistance and described the inheritance of the herbicide resistance. However, no studies have examined genome-wide gene expression changes in response to herbicide treatment in herbicide resistant and susceptible waterhemp.

**Results:**

We conducted RNA-sequencing (RNA-seq) analyses of two waterhemp populations (HPPD-herbicide resistant and susceptible), from herbicide-treated and mock-treated leaf samples at three, six, twelve, and twenty-four hours after treatment (HAT). We performed a de novo transcriptome assembly using all sample sequences. Following assessments of our assembly, individual samples were mapped to the de novo transcriptome allowing us to identify transcripts specific to a genotype, herbicide treatment, or time point. Our results indicate that the response of HPPD-herbicide resistant and susceptible waterhemp genotypes to HPPD-inhibiting herbicide is rapid, established as soon as 3 hours after herbicide treatment. Further, there was little overlap in gene expression between resistant and susceptible genotypes, highlighting dynamic differences in response to herbicide treatment. In addition, we used stringent analytical methods to identify candidate single nucleotide polymorphisms (SNPs) that distinguish the resistant and susceptible genotypes.

**Conclusions:**

The waterhemp transcriptome, herbicide-responsive genes, and SNPs generated in this study provide valuable tools for future studies by numerous plant science communities. This collection of resources is essential to study and understand herbicide effects on gene expression in resistant and susceptible weeds. Understanding how herbicides impact gene expression could allow us to develop novel approaches for future herbicide development. Additionally, an increased understanding of the prolific traits intrinsic in weed success could lead to crop improvement.

**Electronic supplementary material:**

The online version of this article (10.1186/s12870-019-1795-x) contains supplementary material, which is available to authorized users.

## Background

Over the past 30 years, waterhemp (*Amaranthus tuberculatus* (Moq.) J.D. Sauer) has evolved into a major problem weed species in agricultural production systems across the Midwestern United States [1]. If not properly managed, fields infested with waterhemp can suffer yield losses up to 74% in maize (*Zea mays* L.) and 56% in soybean (*Glycine max* (L.) Merr.) [2, 3]. Waterhemp is native to the Midwestern United States and is dioecious; the male and female reproductive structures are on separate plants. As a dioecious species, waterhemp is an obligatory outcrosser, resulting in high levels of genetic recombination and variability. Obligatory outcrossing facilitates the movement of ecologically valuable traits, such as herbicide resistance, among and between waterhemp populations. Additional biological traits that contribute to the weediness of waterhemp include prolific seed production [4], extended and opportunistic germination [5], and rapid growth rate [6].

Herbicides are currently the most important tool in weed management for most crop production systems in many parts of the world [7]. A major concern of modern weed management is evolved resistance to herbicides. One of the first documented cases of evolved herbicide resistance in weeds was reported in 1970 and since then, the number of unique cases (an individual species x specific herbicide site of action) has grown to 498 globally and continues to increase [8, 9].

In 2011, two waterhemp populations with evolved resistance to 4-hydroxyphenylpyruvate dioxygenase (HPPD, EC 1.13.11.27) inhibiting herbicides, including mesotrione, were discovered in seed maize fields in Iowa and Illinois [10, 11]. Mesotrione (2-(4-Mesyl-2-nitrobenzoyl)-1,3-cyclohexanedione, Herbicide Group (HG) 27) is a selective herbicide that is commonly used for broadleaf weed control in maize [12]. HPPD converts 4-hydroxyphenylpyruvate (4-HPP) to homogentisate (2,5-dihydroxyphenylacetate; HGA), which is an important precursor in carotenoid biosynthesis. The herbicidal activity of mesotrione is characterized by the bleaching of new tissue followed by tissue necrosis. While the resistance mechanisms in the Iowa population have not been determined, in the Illinois population, herbicide resistance was conferred by the metabolism of mesotrione to non-herbicidal metabolites, reportedly attributable to increased cytochrome P450 monooxygenase (CYP P450, EC 1.14.14.1) activity [13]. In 2017, mesotrione resistance was confirmed in two waterhemp populations from Nebraska (Columbus, NE, [14] and Tarnov, NE, [15]). In the Tarnov population, which was used in this study, Kaundun et al. [15] found the HPPD gene had no target site mutations associated with mesotrione resistance, nor was the HPPD gene duplicated or overexpressed. However, they observed increased mesotrione metabolism in the resistant population, again attributed to cytochrome P450 activity. Finding similar resistance mechanisms in distant populations (Nebraska and Illinois) suggests resistance occurs through spontaneous evolution of standing genetic variation [16]. Further, in the HPPD-resistant populations examined thus far [17–19], resistance is polygenic, making identification of causal genes more difficult. None of these studies examined genome wide expression changes in response to mesotrione in resistant and susceptible waterhemp populations. Characterizing gene expression differences in HPPD-resistant and susceptible waterhemp populations at time points well before metabolic responses are detected could help identify major genes contributing to resistance and may provide insight into managing the evolution of resistance to other herbicides in waterhemp and possibly other weed species.

Advances in sequencing technologies have created opportunities to study the genomics of non-model organisms [20]. Due to a lack of weed-related genomic resources, Lee et al. [20] sampled 43 million base pairs of the waterhemp genome using 454 pyrosequencing (Roche Sequencing, Pleasanton, CA, USA). While this sequencing approach covered less than 10% of the waterhemp genome, it demonstrated that cutting-edge sequencing technology could be applied to weed species. Riggins et al. [21] used 454 pyrosequencing to analyze the waterhemp transcriptome. To maximize transcriptome coverage, the study pooled RNA samples from different individuals, sexes, tissues, life stages, herbicide treatments, and cold stress. These studies contributed to a better understanding of the waterhemp genome and provided sequence-based details for key enzymes targeted by herbicides and potentially prone to herbicide resistance evolution [21]. However, the experimental designs and sequencing platforms used in these studies made it impossible to identify the genes and gene networks that regulate susceptibility, tolerance and resistance to herbicides. Since these initial studies, RNA-sequencing (RNA-seq) has become the standard method for transcriptome analyses for species lacking genomic resources.

The increasing prominence of waterhemp as an economically important and ubiquitous weed in the Midwestern United States and the demonstrated ability to evolve resistance to herbicides makes this species an important model for studying herbicide resistance evolution in weeds. Here we report on the sequencing of the waterhemp transcriptome using high throughput RNA-seq technology. This study identifies the genes and gene networks responding to the HPPD-inhibiting herbicide mesotrione in susceptible and resistant waterhemp genotypes over a 24-h exposure time course. In addition, our study provides a publicly available sequence-based platform for the weed science community to study this agronomically important weed.

## Methods

### Tissue collection

Two waterhemp populations with different susceptibility phenotypes to HPPD-inhibiting herbicides (susceptible and resistant) were selected. The susceptible waterhemp population was collected from the Curtis Farm at Iowa State University (Ames, IA, USA) in 2006. The resistant population was from Tarnov, Nebraska (USA) and was collected in 2014 from a field with a history of seed maize production after reports of waterhemp surviving multiple applications of mesotrione [15].

Each genotype was planted in 40 individual 15.2 cm in diameter round pots using a 4:1 ratio of Sunshine potting mix #1/LC1 (Sun Gro Horticulture, Agawam, MA, USA) to sand, respectively. We added 1 tsp. of Osmocote Flower Food Granules (14–14-14) (The Scotts Miacle-Gro Company, Marysville, OH, USA) to each pot at the time of planting. Plants were grown in a greenhouse set to 24 °C with a 14-h photoperiod supplemented by high-pressure sodium bulbs. Plants were watered every other day. After 2 weeks, seedlings were thinned to 3 plants per pot. Each plant within each pot was randomly assigned a label of A, B, or C. The pots were placed in the greenhouse in a randomized block.

When plants reached a minimum height of 7.6 cm, they were treated with mesotrione applied in a CO_2_ powered spray chamber equipped with TeeJet® 80015EVS nozzles (Spraying Systems Co., Wheaton, IL, USA) at a carrier volume of 191.76 L ha^− 1^. Half of each population (20 pots of each genotype) was treated with 105.36 g ai ha^− 1^ of mesotrione, 1% (v/v) crop oil concentrate (COC), 2.5% (v/v) urea (CH_4_N_2_O) ammonium nitrate (NH_4_NO_3_) solution (UAN; 28% nitrogen), and tap water. The other half was treated with water, representing a mock treatment. The plants were then returned to the greenhouse into 4 blocks separated by treatment and genotype. To determine other reagents used in conjunction with the herbicide affected plant growth, a separate study was used to evaluate the phenotypic response of plants that were untreated, mock-treated, and treated with 1% (v/v) COC, 2.5% (v/v) UAN (28% nitrogen), and tap water (data not provided). No difference in phenotypic response was identified.

Each genotype within a treatment was separated into 4 groups of 5 pots. The 4 groups were randomly assigned a time point of 3, 6, 12, or 24 HAT. Within each time point 4 pots were labeled 1–4. The fifth pot was used as a control for verification of the phenotypic response and was also used as a buffer against greenhouse variation in the bench space adjacent to the wall. Leaf tissue from each plant within the four labeled pots was collected at 12:00 PM CDT (3 HAT), 3:00 PM CDT (6 HAT), 9:00 PM CDT (12 HAT), and 9:00 AM CDT (24 HAT) on May 22 and 23 of 2015. Sunrise occurred at 5:49 AM, while sunset occurred at 8:34 PM. A given plant was only sampled at one time point. To obtain the highest quality RNA, the four youngest fully-developed leaves of each plant were excised at the base of each leaf, placed in a 50 mL Falcon® tube (Thermo Fisher Scientific, Waltham, MA, USA), flash frozen in liquid nitrogen, and then maintained at − 80 °C. Tissues from individual plant samples were stored in a separate Falcon® tube. Plants continued to grow for 3 weeks after treatment to verify the phenotypic response to mesotrione.

### RNA isolation

Frozen tissue in the 50 mL Falcon® tubes was crushed by inverting an 11.11 cm pestle, dipped in liquid nitrogen, into the tubes. Crushing the leaf samples within a Falcon® tube mixed the tissue from an individual plant providing a more homogeneous collection of leaves from each plant. One full microspatula scoop (approximately 100 mg) of crushed frozen tissue from each Falcon® tube was added to a 2 mL Safe-Lock™ microcentrifuge tube (Eppendorf, Hamburg, Germany) kept on dry ice with a 3 mm tungsten carbide bead. Prepared microtubes were placed in TissueLyser Adapter sets precooled at − 80 °C and then processed in a Qiagen TissueLyser II (Qiagen, Valencia, CA, USA) for 1 min at 30 Hz. RNA extraction was performed as recommended by the manufacturer using the RNeasy® Plant Mini Kit (Qiagen, Valencia, CA, USA). To check for RNA concentration and quality, a NanoDrop™ 1000 Spectrophotometer (Thermo Fisher Scientific, Waltham, MA, USA) was used.

Prior to DNase treatment, to remove genomic DNA contamination, RNA samples from plants growing in the same pot were pooled together. 6 μg of pooled RNA (2 μg of RNA per sample) were used in a 50 μL DNase reaction using the Ambion® TURBO DNA-*free*™ Kit (Thermo Fisher Scientific, Waltham, MA, USA). Immediately after DNase treatment, samples were further purified using the RNeasy® MinElute® Cleanup Kit following the manufacturer’s recommendations. RNA concentration and quality of the samples were checked using the NanoDrop™ 1000 Spectrophotometer.

### RNA-Seq and de novo transcriptome assembly

The extracted RNA was sequenced by the Iowa State University DNA Facility using the Illumina HiSeq 2500 (Illumina, Inc., San Diego, CA, USA) platform. Prior to sequencing, the quality of all samples was confirmed using an Agilent® 2100 Bioanalyzer™ (Agilent®, Santa Clara, CA). RNA was considered acceptable if the RNA integrity number (RIN) was greater than seven. Sequences were generated in High Output Mode with 100 base pair read length and paired-end sequencing. The paired-end protocol, sequencing the RNA from both directions of the strand, enables better transcriptome coverage. Forty-eight samples were run on one eight-lane flow cell, six samples per lane. Each lane contained three samples of each treatment (herbicide or mock), of one waterhemp genotype (resistant or susceptible), at one time point (3, 6, 12, or 24 HAT).

The programs Scythe [22] and Sickle [23] were used to remove sequencing artifacts, low-quality bases (q < 20) and short reads (l < 50) from all 48 sequenced samples. Trinity (version 2.0.6, [24]) was used to produce multiple de novo transcriptome assemblies. Three separate assemblies (versions 1–3) were made using kmer lengths of 25, 29, and 32 (Additional file 1). After comparing assembly statistics (total number of transcripts, contig N50, median contig length, and average contig size), version 3 (kmer length 32) was selected because this assembly resulted in the longest N50 (Table 1). However, assembly version 3 still contained contigs that lacked open reading frames or were expressed at very low levels. Therefore, in order to create an improved assembly that could be used for measuring differential gene expression, this assembly was processed with three additional steps. First, the TransDecoder utility within Trinity [25] was used to return transcripts that contained an open reading frame (ORF) of at least 100 amino acids. Second, the program kallisto [26] was used to estimate the number of counts per transcript. Any transcripts with less than 10 counts were removed. Finally, BLASTN analyses (E-value cutoff of E < 10^− 20^, [27]) was used to compare the v3 assembly to predicted transcripts in the sugar beet genome (*Beta vulgaris* L., Refbeet v1.2, [28]), representative species of the ten plant clades of Phytozome (version 10, [29]), and all sequences available in the GenBank nucleotide (NT) database (version 1/22/2016, [30]). Any transcript that was best matched to a non-plant species or had no significant hits was not included in the final assembly. These filtering steps resulted in the final transcriptome, version 4 (v4, Additional file 2).

**Table 1:** Comparison of waterhemp transcriptome assemblies

		v1 (k-mer = 25)	v2 (k-mer = 29)	v3 (k-mer = 32)	v4
	Total ‘gene’ count	269,388	238,782	226,402	42,040
	Total transcript count	512,945	471,767	451,199	113,893
All Transcripts	Contig N50	762	926	1029	1709
	Median Contig Length	390	424	448	1094
	Average Contig Length	598.54	669.21	713.94	1317.45
Longest Isoform	Contig N50	662	747	792	1816
	Median Contig Length	359	371	375	998
	Average Contig Length	549.32	590.09	609.47	1301.70

Trinity (version 2.0.6, [24]) was used to generate three unique de novo waterhemp transcriptome assemblies: v1, v2, and v3 based on different kmer length requirements. With each assembly, the number of total transcripts decreased while average contig length increased (see Additional file 1). A total of 2.3 billion reads representing different genotypes, treatments and time points were used in the assembly. The v4 assembly is a subset of the v3 assembly, with transcripts that were redundant, lacking open reading frames or expressed at low levels removed

### Functional annotation

The v4 transcriptome was annotated (Additional file 3) using BLASTX (E < 10^− 10^, [27]) against proteins from *Arabidopsis thaliana* (The Arabidopsis Information Resource version 10 [TAIR10], [31]), sugar beet (Refbeet v1.2, [28]) and Uniref100 (version 1/22/2016, [32]) and using BLASTN (E < 10^− 20^, [27]) against nucleotides from grain amaranth (Phytozome v12.1, *Amaranthus hypochondriacus* v2.1, [33]). Custom Perl scripts were used to assign gene ontology (GO) biological processes and molecular function terms [34] based on the top *A. thaliana* hit. To measure the breadth of the de novo v4 transcriptome relative to related species with complete genome sequences, predicted proteins from poplar (Phytozome v12.1.6, *Populus trichocarpa* v3.1, [35]), papaya (Phytozome v12.1.6, *Carica papaya ASGPBv0.4*, [36]), asparagus (Phytozome v12.1.6, *Asparagus officinalis V1.1*, [37]), sugar beet (Refbeet v1.2, [28]), and grain amaranth (Phytozome v12.1, *Amaranthus hypochondriacus* v2.1, [33]) genomes were also compared to *A. thaliana* (TAIR version 10, [31]) using BLASTP (E < 10^− 10^, [27]). Custom Perl scripts were then used to assign GO biological process and GO slim information based on the best *A. thaliana* homolog. Within each species, the total number of each GO slim count was divided by the total count of all GO slims to adjust for genome duplications.

### Differential expression analyses

The individual sample reads were mapped to the version 4 transcriptome assembly using Bowtie [38]. RNA-seq by Expectation-Maximization (RSEM) software [39] was used to account for reads that could re-align to multiple assembled transcripts in the de novo assembly due to alternatively spliced isoforms. The raw expression counts were normalized across samples using the Trimmed Mean of M-values (TMM) method [40] in edgeR [41]. GGplot2 (CRAN, [42]) was used to compare and visualize read counts across replicate samples for technical reproducibility. Transcripts with a log count per million less than one (log CPM < 1) across all samples were excluded from the analyses, leaving 72,697 expressed transcripts (v4 isoforms). edgeR was also used to identify significantly (false discovery rate (FDR) < 0.05, [43]) differentially expressed transcripts (DETs, Table 2, Additional file 4) responding to treatment (mesotrione treatment vs. mock) in each genotype at each time point (R3, R6, R12, R24, S3, S6, S12 and S24) and across all time points (R and S).

**Table 2:** Summary of the differentially expressed transcripts (DETs) responding to mesotrione treatment in waterhemp

Genotype	Hours After Treatment				
	3	6	12	24	Across Time
Resistant	89	62	61	1983	2091
Susceptible	500	77	61	565	1246
Overlap	42	5	9	83	330
Percent Overlap	7.7%	3.7%	8.0%	3.4%	11.0%

DETs were identified at 3, 6, 12, and 24 h after treatment (HAT) in the resistant and susceptible waterhemp genotypes. Percent overlap between genotypes at a specific time point was calculated by dividing the number of DETs in common between genotypes by the total number of unique DETs at

Overrepresented GO terms associated with DETs of interest were identified using a Fisher’s exact test [44] to compare the number of times each GO term was found within a list DETs of interest relative to the number of times each GO term was found among all transcripts in the v4 assembly (Additional file 5). A Bonferroni correction (*P* < 0.05, [45]) was applied to correct for over testing.

### Clustering of herbicide-responsive DETs

To determine if herbicide-responsive DETs might physically cluster in the genome, we took advantage of the closely related grain amaranth genome [33]. BLASTN (E < 10^− 20^, [27]) was used to compare waterhemp transcripts to predicted transcripts from the grain amaranth genome (Phytozome v12.1, *Amaranthus hypochondriacus* v2.1, [33]). BLASTN was then used to compare the top grain amaranth hit (E < 10^− 20^, [27]) back to all waterhemp transcripts. Waterhemp transcripts were considered orthologous to grain amaranth transcripts if reciprocal BLAST identified any of the original waterhemp transcript isoforms as a top hit. Custom Perl scripts were used to identify the genomic location of the orthologous grain amaranth transcript from the general feature format (GFF) file corresponding to grain amaranth genome.

A window size of 100,000 bp, centered on transcription start sites, was used to identify clusters of DETs. DETs that overlapped by ±50,000 bp from transcription start sites were considered part of the same cluster. Start site positions from transcripts in the same cluster were used to calculate an average position for the cluster. Only clusters with four or more DETs are reported (Additional file 6). For clustering, multiple isoforms mapping to the same cluster were considered a single DET transcript.

### Identification of candidate single nucleotide polymorphisms between resistant and susceptible waterhemp genotypes

The sequence alignment files generated for differential expression analysis were sorted using samtools and then merged by genotype into two master files. The samtools pipeline was used to identify biallelic single nucleotide polymorphisms (SNPs) relative to the v4 assembly. The samtools output was filtered to identify SNPs between the resistant and susceptible genotypes and to only include SNPs with a minimum Phred-scaled probability score of QUAL ≥25 and homozygous within a genotype (GT) but unique to each genotype (Additional file 7). Genotype likelihoods (PL ≥ 200 and PL = 0; Phred-scaled data likelihoods of possible genotypes) were used to increase the confidence of reported genotypes for reported SNPs. As RNA samples were pooled from multiple resistant or susceptible plants, only SNPs with at least 90% of a single allele in the resistant and susceptible populations are reported. To predict the relative location of SNPs in the waterhemp genome, we again used the grain amaranth genome, taking advantage of the reciprocal BLASTN (E < 10^− 20^, [27]) described previously.

## Results

### Phenotypic assessment of mesotrione responses in resistant and susceptible waterhemp genotypes

**Fig. 1:**
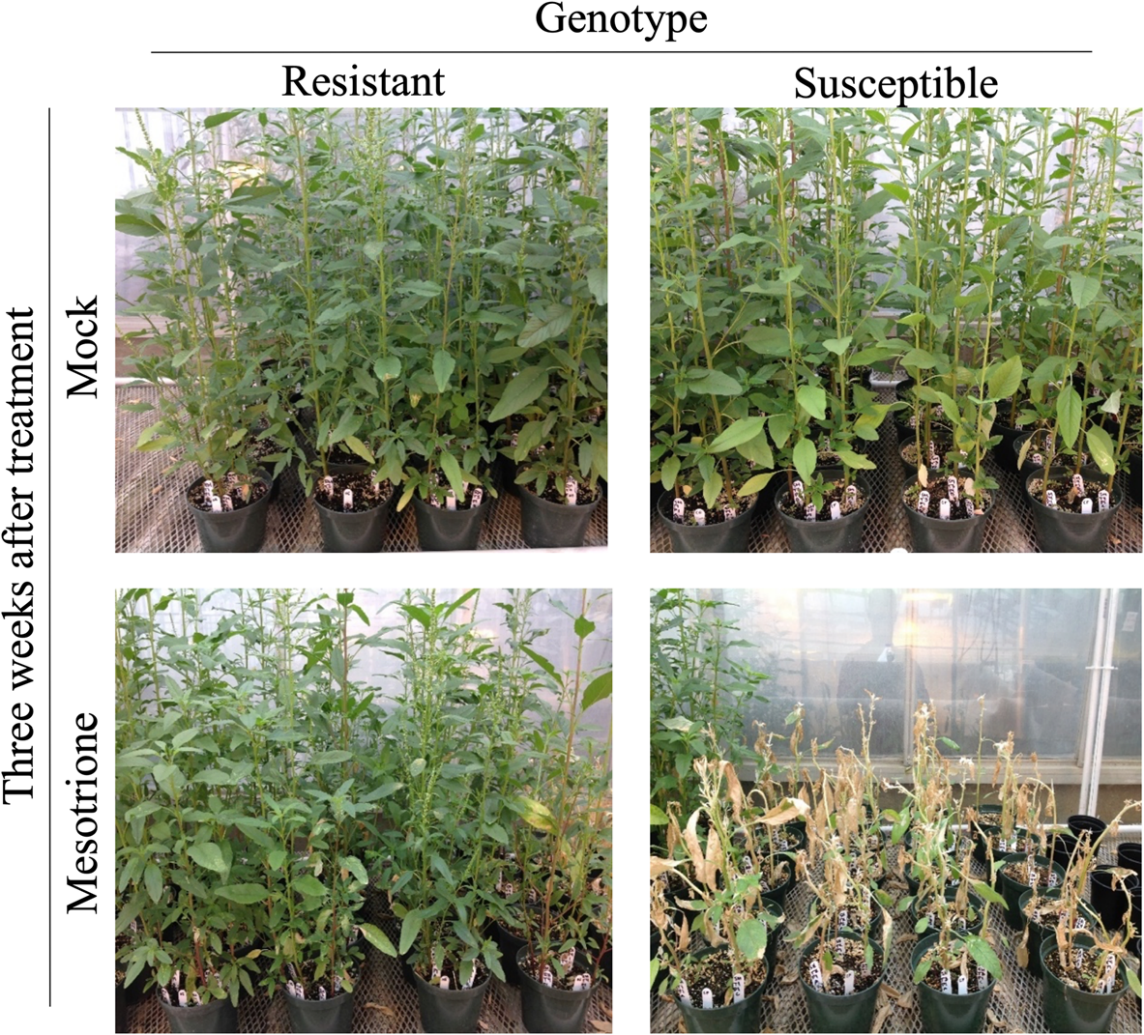
Phenotyping for mesotrione resistance. To confirm that the waterhemp samples used for RNA-seq analyses were properly treated, an additional set of plants was grown simultaneously with the plants sampled for RNA-seq. No tissues were collected from these plants, instead they were allowed to continue growing for three additional weeks after treatment to assess mesotrione herbicide injury. All plants exhibited the expected phenotype

Samples used for RNA-seq were harvested prior to the development of visual mesotrione treatment symptoms; therefore, herbicide-treated and mock-treated control plants were maintained in the greenhouse for 3 weeks after mesotrione application to assess phenotype responses. Both genotypes responded to the mesotrione application as expected. The resistant population initially displayed the major HPPD-inhibiting herbicide characteristics of chlorosis and bleached meristematic growth followed by necrosis but recovered by the third week after application. Visual comparison of mock-treated resistant and mock-treated susceptible to mesotrione-treated resistant (Fig. 1) at 3 weeks after treatment showed slight differences, primarily minor stunting and sparse tissue damage within the canopy of the mesotrione-treated resistant population. Conversely, the mesotrione-treated susceptible population sustained heavy tissue bleaching and eventually necrosis and plant death. These observations and comparisons verified the proper herbicide response of both genotypes to mesotrione treatment.

### RNA-Seq and de novo assembly of the waterhemp transcriptome

Purified RNA from three replicates of 16 samples (3, 6, 12, and 24-h samples of mesotrione-treated or mock-treated susceptible and resistant genotypes) were sent to the Iowa State University DNA Facility for the creation and sequencing (100 base pair, paired-end sequencing) of 48 multiplex libraries. A total of 2.45 billion raw reads were produced. Following the sequence clean up described in the Materials and Methods, 2.36 billion sequences were used for de novo transcript assembly using the program Trinity (version 2.0.6, [24]) with three different kmer lengths (k = 25, 29, and 32). Sequences from all samples were used to yield a broad representation of the waterhemp transcriptome and allow the identification of genes expressed in a genotype, treatment or time-specific manner.

When comparing the three assemblies (v1, v2 and v3) generated with differing kmer lengths (25, 29, and 32, respectively) we noted that as the kmer length increased, transcript number decreased and Contig N50 increased (Table 1). The Contig N50 is a weighted median of contig (contiguous overlapping sequences) length where 50% of the assembled nucleotides are contained in contigs greater than or equal to the length of the Contig N50; it can be used as an important measurement in assembly evaluations and was a major factor in the decision of which assembly to use for our analysis [46, 47]. In addition, we visualized contig length distribution for each of our different assemblies (Additional file 1). As suggested by the contig statistics, increasing kmer size increased average contig length and decreased the number of contigs. This was especially evident for contigs smaller than 1000 base pairs (Log_10_ 3). Therefore, we chose to focus on the third assembly (v3, kmer = 32) for subsequent analysis. Following selection of the v3 assembly, we still needed to remove sequences that lacked open reading frames (ORFs), were redundant, or were expressed at extremely low levels. From the initial v3 assembly containing 451,199 transcripts, TransDecoder [25] was used to identify all transcripts with ORFs> 100 base pairs and remove redundant transcripts, leaving 128,737 transcripts. Similarly, kallisto [26] identified 97,944 lowly-expressed transcripts in the v3 assembly. Cross-referencing the TransDecoder and kallisto datasets resulted in 119,635 transcripts with ORFs> 100 bp and read counts > 10. Finally, a series of BLASTN analyses described in the materials and methods were used to eliminate transcripts with the best homology to non-plant species or transcripts with no significant hits. This left 113,893 transcripts as the basis of our de novo waterhemp transcriptome (v4) used for differential expression analyses. Sequences for the v4 assembly can be found in Additional file 2.

### Functional annotation of the waterhemp transcriptome

The waterhemp v4 assembly was annotated using BLASTX (E < 10^− 10^_,_ [27]) against predicted proteins from *A. thaliana* (The Arabidopsis Information Resource version 10 [TAIR10], [31]), sugar beet (Refbeet v1.2, [28]) and Uniref100 (version 1/22/2016, [32]) and using BLASTN (E < 10^− 20^, [27]) against predicted transcripts from grain amaranth (Phytozome v12.1, *Amaranthus hypochondriacus* v2.1, [33]). The best *A. thaliana* hits were used to assign the gene ontology (GO) biological processes and the molecular function terms [34] to each transcript of the v4 assembly. Annotations for the v4 assembly can be found in Additional file 3.

**Fig. 2:**
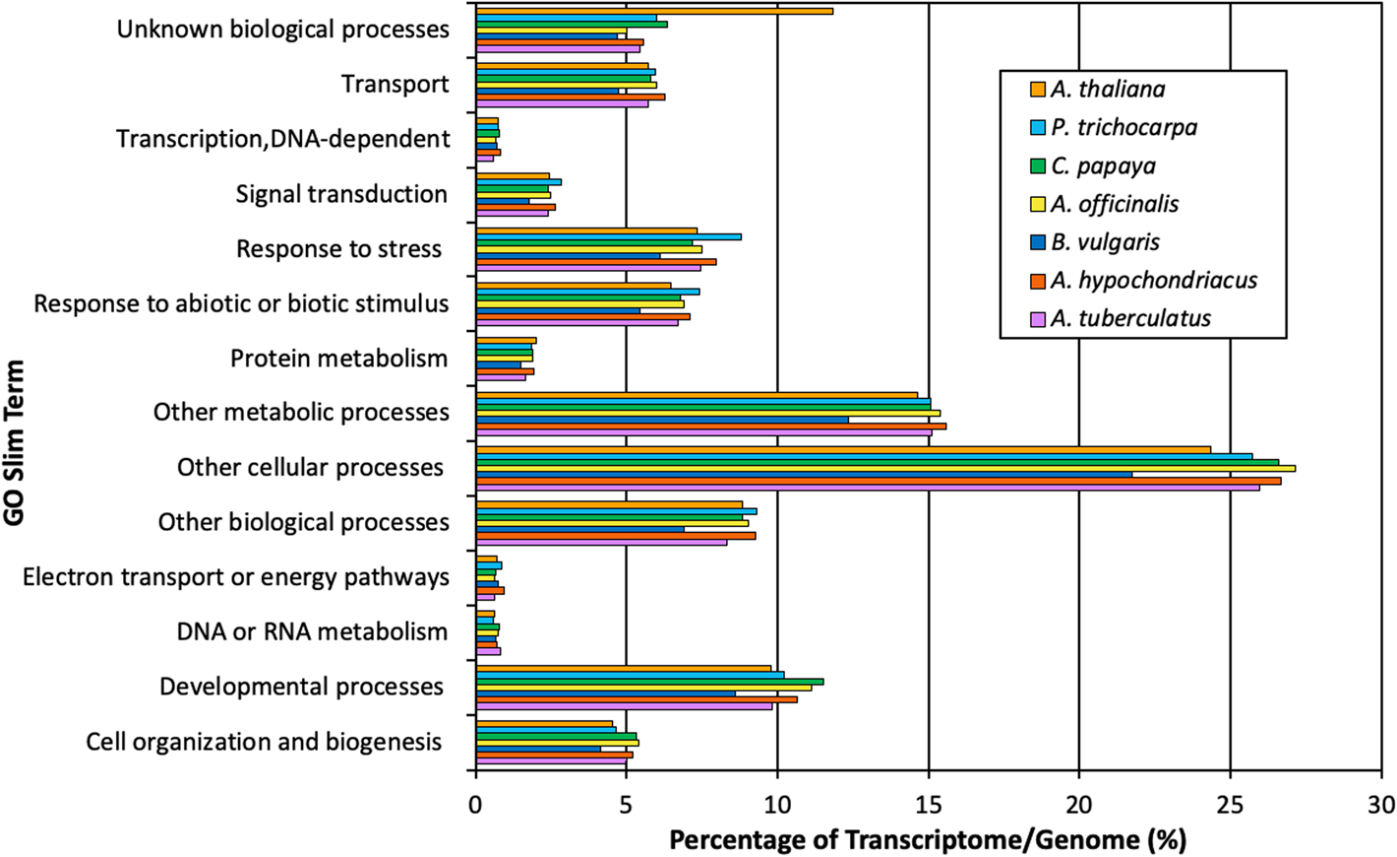
Comparing the breadth of the waterhemp v4 transcriptome to genomes of related species. Gene ontology biological process (GOBP) slim terms were used to compare the breadth of the waterhemp v4 transcriptome relative to predicted proteins from the *Populus trichocarpa*, *Carica papaya*, *Asparagus officinalis*, *Beta vulgaris*, *Amaranthus hypochondriacus*, and *Arabidopsis thaliana* genomes. The percentage of annotations associated with each GOBP slim term is consistent across all seven species, confirming the waterhemp v4 transcriptome is a suitable proxy to use in the absence of a genome sequence

To verify the accuracy and coverage of the v4 assembly, GO biological process terms inferred from homology with *A. thaliana* were mapped to GO slim terms using custom Perl scripts. GO slim term abundance was then compared between the waterhemp v4 transcriptome assembly and all predicted proteins of the *A. thaliana*, poplar, papaya, asparagus, sugar beet, and grain amaranth genomes (Fig. 2). Waterhemp, sugar beet, and grain amaranth all belong to the Amaranthaceae family [28, 33], poplar, papaya and asparagus are dioecious species [35–37], and *A. thaliana* is a well-established plant model [48]. For each GO slim term, the abundance of assigned transcripts was measured as a percentage relative to the entire transcriptome or genome, allowing us to normalize for any potential genome duplications within a given species. We found that for thirteen of the fourteen GO slim terms, the v4 waterhemp transcriptome assembly was comparable to the genomes of the six other species. This suggests the breadth of the waterhemp v4 transcriptome is consistent with the breadth of the *A. thaliana*, poplar, papaya, asparagus, sugar beet, and grain amaranth genomes. The only exception was the GO slim term ‘unknown biological process’ which was overrepresented in *A. thaliana*, compared to the six other species.

### Identification of waterhemp transcripts differentially expressed in response to mesotrione

To allow identification of DETs responding to mesotrione treatment in each genotype, individual samples were mapped to the v4 waterhemp assembly using the protocol described in the Trinity user manual [24]. In total, 782,456,581 reads were mapped to the assembly. The raw expression counts were normalized across samples using the Trimmed Mean of M-values (TMM) method [40] in edgeR [41]. Following visual inspection, all replicate samples were considered good quality. Isoforms were considered expressed if they contained at least 1 count per million across three samples or replicates. Of the 113,893 isoforms in the v4 assembly, 72,697 were considered expressed (v4 isoforms). The average length for the expressed isoforms was 1580 base pairs and contigs assumed a normal distribution (Additional file 1).

edgeR was used to identify DETs responding to mesotrione treatment relative to mock-treated controls within each genotype (herbicide resistant and susceptible) across time and at specific time points (3, 6, 12, and 24 h after treatment (HAT)). DET expression is reported as a log_2_ fold change (log_2_ FC). A log_2_ FC greater than 1 indicates a DET is induced by the mesotrione treatment, while a log_2_ FC less than one indicates a DET is repressed by the mesotrione treatment. DETs with an FDR < 0.05 [43] are considered significantly differentially expressed in response to mesotrione treatment (Additional file 4).

**Fig. 3:**
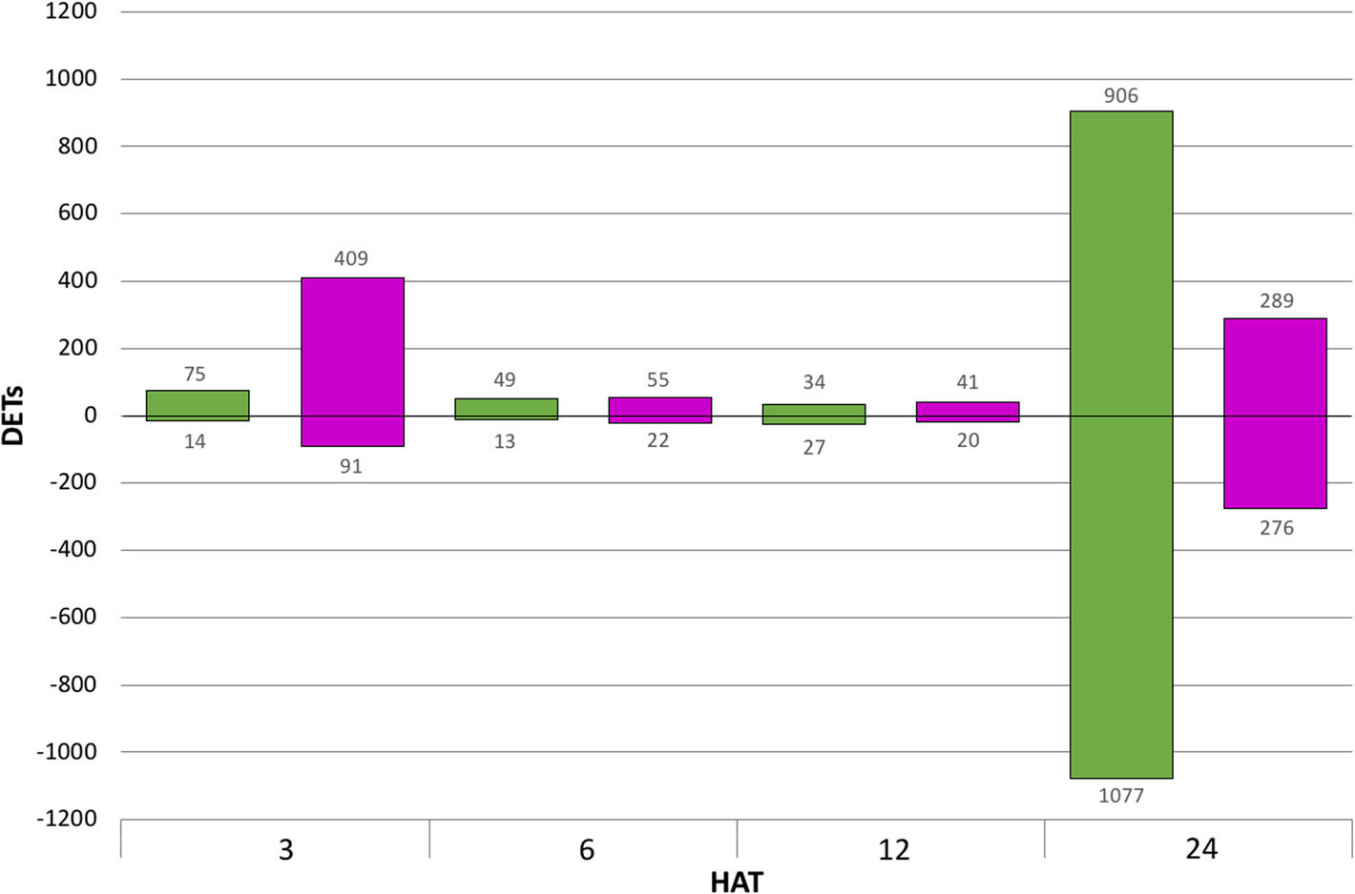
Identification of waterhemp differentially expressed transcripts (DETs) responding to mesotrione treatment across time. To identify DETs at each time point (3, 6, 12, and 24 h after treatment (HAT)), transcript expression in resistant (green) or susceptible (magenta) genotypes treated with mesotrione was compared to mock-treated controls. The values above and below the bars represent the number of DETs that were induced and repressed, respectively

**Fig. 4:**
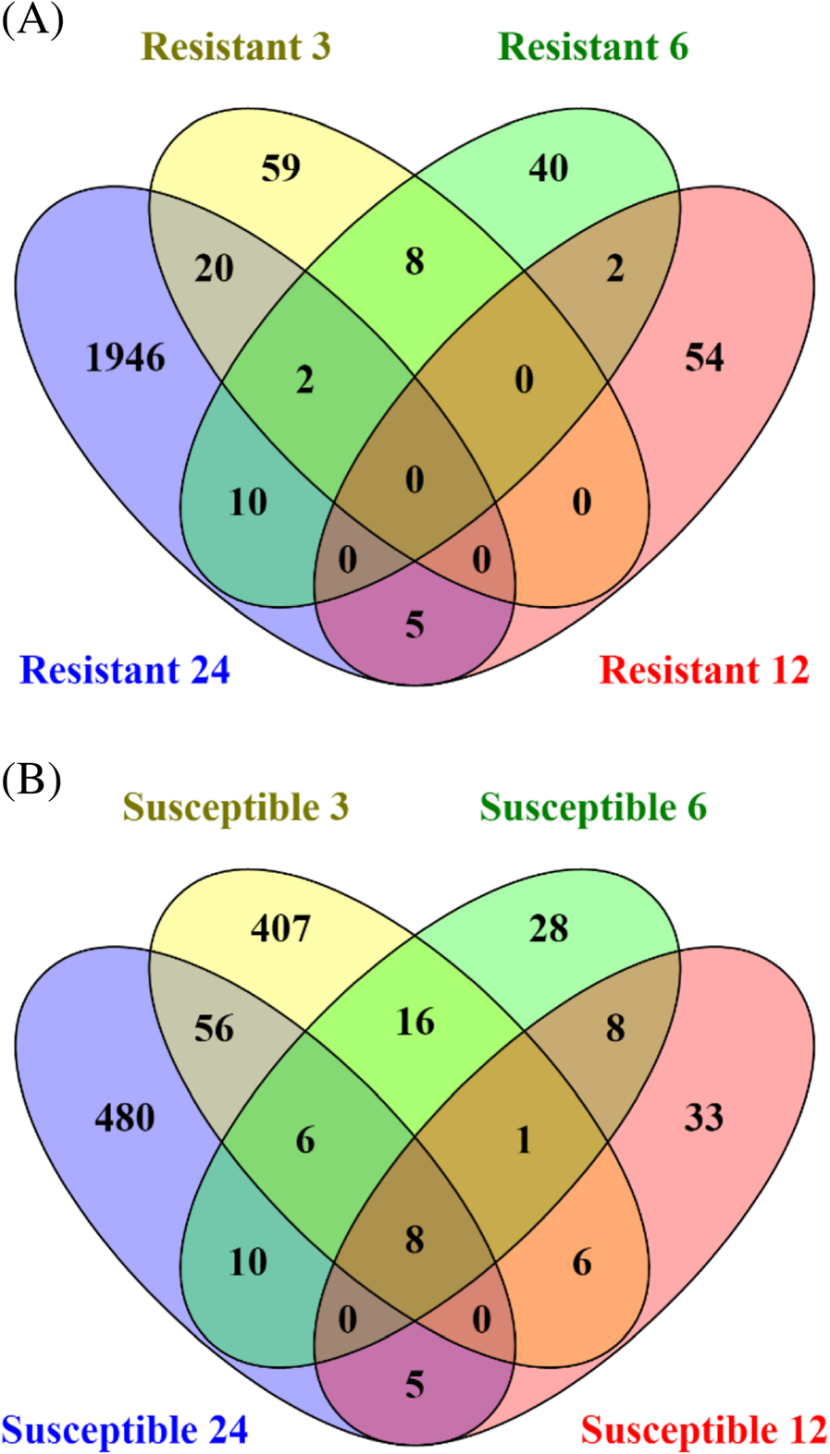
Mesotrione responsive differentially expressed transcripts (DETs) show little overlap between timepoints. **a** Comparison of DETs in the resistant genotype at 3, 6, 12, and 24 h after treatment (HAT). **b** Comparison of DETs in the susceptible genotype at 3, 6, 12, and 24 HAT

We identified 89, 62, 61, and 1983 DETs in the resistant waterhemp genotype at 3, 6, 12, and 24 HAT, respectively, and 500, 77, 61, and 565 DETs were identified in the susceptible waterhemp genotype at 3, 6, 12, and 24 HAT, respectively (Table 2). We plotted the number of DETs per genotype within each time point to analyze expression trends across time (Fig. 3). The susceptible waterhemp genotype exhibited large fluxes in DET expression across time. At 3 HAT the susceptible genotype induced 409 transcripts suggesting a quick initial response to the mesotrione treatment. The response diminishes in the middle two time points but then increases again at 24 HAT. In contrast, the resistant waterhemp genotype demonstrated little response to mesotrione treatment at 3, 6 and 12 HAT while a large number of transcripts respond at 24 HAT. Remarkably, while symptoms in response to HPPD herbicide treatments can take as long as 1 week to develop, both resistant and susceptible waterhemp genotypes responded within three HAT. Furthermore, few DETs overlapped between time points within a given genotype (Fig. 4) or between genotypes (Table 2). At 3, 6, 12, and 24 HAT we found 7.7, 3.7, 8, and 3.4% of DETs were common to both waterhemp genotypes, respectively, suggesting a rapid and dynamic response to mesotrione treatment (Table 2).

In addition to identifying transcripts responding to mesotrione treatment at specific time points, we also identified transcripts responding to mesotrione treatment across time. We identified 2091 and 1246 DETs responding to mesotrione treatment across time in the resistant and susceptible genotypes, respectively (Additional file 4). Of these, only 330 DETs were common to both waterhemp genotypes. This reaffirms that the resistant and susceptible genotypes have different responses to the mesotrione treatment.

### Characterization of mesotrione responsive transcripts

While differential expression is useful in identifying individual transcripts found in response to the mesotrione treatment, we were interested in identifying transcripts responding to mesotrione treatment that might have similar functions or act in the same molecular pathway. Therefore, for each time point by genotype combination, we used a Fisher’s Exact Test [44] with a Bonferroni correction [45] to identify gene ontology biological process terms [49] significantly overrepresented (*P* < 0.05) among DETs, relative to the waterhemp v4 assembly (Additional file 5). In the resistant waterhemp genotype, we identified 11 and 12 GO terms significantly overrepresented at 3 and 24 HAT. No significant GO terms were identified at 6 and 12 HAT. Combining all DETs from the resistant waterhemp genotype, we identified 18 significantly overrepresented GO terms. In the susceptible waterhemp genotype, we identified 34, 3, 2 and 24 significant GO terms at 3, 6, 12 and 24 HAT, respectively. Combining all DETs from the susceptible waterhemp genotype, we identified 39 significantly overrepresented GO terms.

**Fig. 5:**
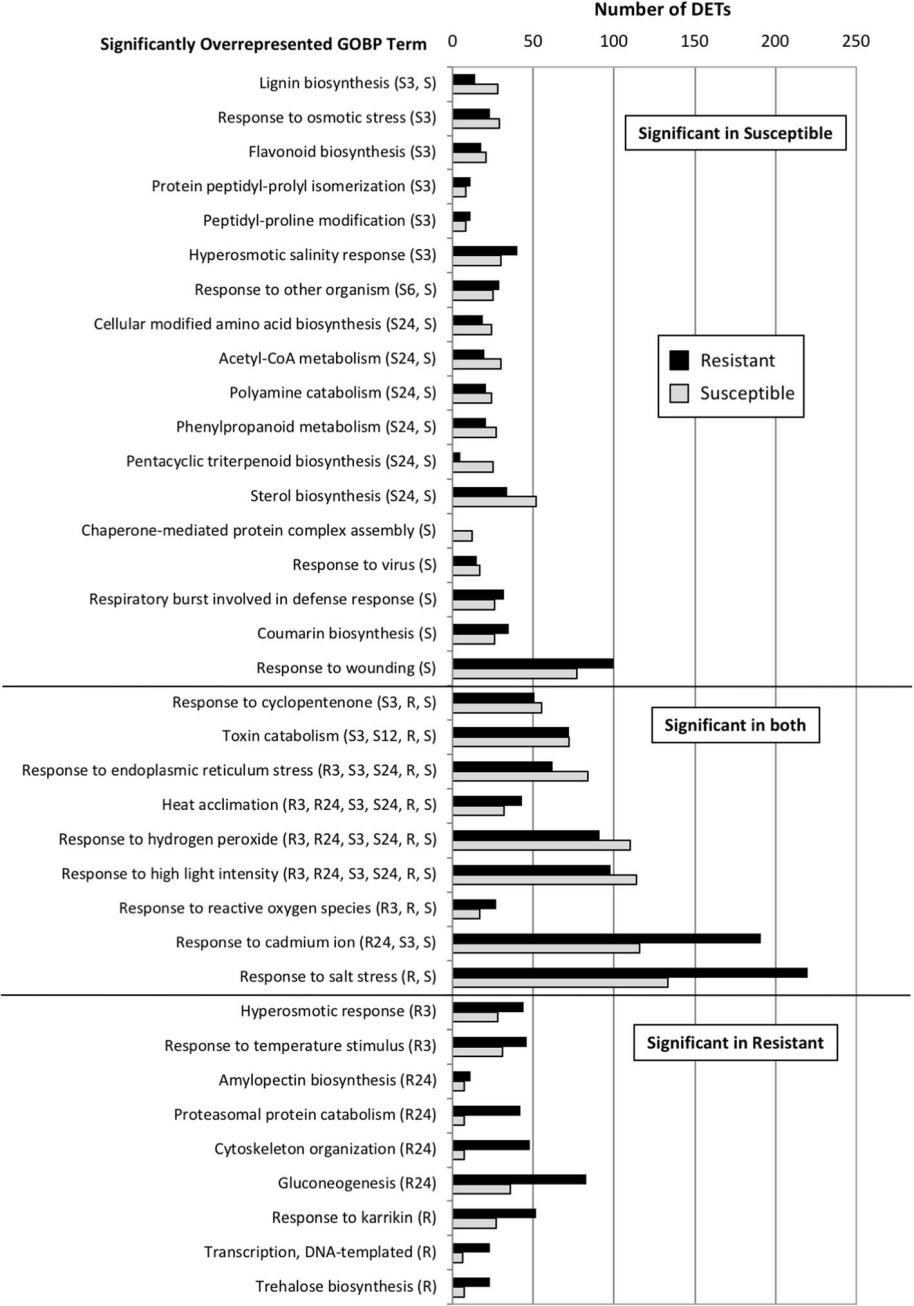
Characterization of mesotrione responsive differentially expressed transcripts (DETs) using gene ontology (GO) overrepresentation. A Fisher’s Exact Test [44] with a Bonferroni correction [45] was used to identify significantly (*P* < 0.05) overrepresented gene ontology biological process terms associated with genotype-specific DETs at a given time point or across time, relative to all transcripts in the waterhemp v4 transcriptome (Additional file 6). For each GO term, all significant time points are indicated in parentheses. To aid in data visualization, GO terms with DETs that perfectly overlapped with a larger, significant GO term were removed. In addition, only GO terms with at least 10 DETs in either the resistant or susceptible genotype are shown. Data is divided to demonstrate significant GO processes unique or common to resistant and susceptible genotypes

To allow direct comparison between resistant and susceptible waterhemp genotypes, we compared unique transcript counts for significant GO terms (P < 0.05) identified at specific time points and over time in both genotypes (Fig. 5). To aid in data visualization, GO terms with DETs that perfectly overlapped with a larger, significant GO term were removed. In addition, only GO terms with at least 10 DETs in either the resistant or susceptible waterhemp genotype are shown. Using this approach, we were able to identify 18 GO terms significantly overrepresented only in the susceptible waterhemp genotype, nine GO terms significantly overrepresented only in the resistant waterhemp genotype and nine GO terms significantly overrepresented in both waterhemp genotypes.

GO terms uniquely overrepresented in the susceptible waterhemp genotype response were largely associated with stress and defense responses including ‘response to osmotic stress’ (GO:0006970), ‘response to hyperosmotic salinity’ (GO:0042538), ‘response to other organism’ (GO:0051707), ‘response to virus’ (GO:0009615), ‘response to wounding’ (GO:0009611), and ‘respiratory burst involved in defense response’ (GO:0002679). Other significantly overrepresented GO terms were associated with metabolism including ‘lignin metabolism’ (GO:0009809), ‘flavonoid metabolism’ (GO:0009813), ‘coumarin metabolism’ (GO:0009805), ‘cellular modified amino acid’ (GO:0042398), ‘pentacyclic triterpenoid biosynthesis’ (GO:0019745), ‘sterol biosynthesis’ (GO:0016126), ‘acetyl-CoA metabolism’ (GO:0006084), ‘phenylpropanoid metabolism’ (GO:0009698), and ‘polyamine catabolism’ (GO:0006598). Other significant GO terms included ‘protein peptidyl-prolyl isomerization’ (GO:0000413), ‘peptidyl-proline modification’ (GO:0018208), and ‘chaperone-mediated protein complex assembly’ (GO:0051131). For 11 of the 18 significantly overrepresented GO terms unique to the susceptible waterhemp genotype, we observed more DETs in the susceptible than the resistant genotype.

GO terms significantly overrepresented in both waterhemp genotypes included ‘response to cyclopentenone’ (GO:0010583), ‘response to endoplasmic reticulum stress’ (GO:0034976), ‘response to hydrogen peroxide’ (GO:0042542), ‘response to high light intensity’ (GO:0009644), ‘response to reactive oxygen species’ (GO:0000302), ‘response to cadmium ion stress’ (GO:0046686), and ‘response to salt stress’ (GO:0009651), ‘heat acclimation’ (GO:0010286), and ‘toxin catabolism’ (GO:0009407). For five of the nine GO terms significant in both genotypes, a greater number of DETs were observed in the susceptible waterhemp genotype.

GO terms uniquely overrepresented in the resistant waterhemp genotype were quite varied in their functions. Similar to the responses in the susceptible waterhemp genotype, we identified GO terms associated with response to stress (i.e., ‘hyperosmotic response’ (GO:0006972), ‘response to temperature stimulus’ (GO:0009266), and ‘response to karrikin’ (GO:0080167)). Interestingly, a number of GO terms were associated with energy metabolism including ‘amylopectin biosynthesis’ (GO:0010021), ‘proteasomal protein catabolism’ (GO:0010498), ‘gluconeogenesis’ (GO:0006094) and ‘trehalose biosynthesis’ (GO:0005992). Other significant GO terms observed in the resistant waterhemp genotype included ‘cytoskeleton reorganization’ (GO:0007010) and ‘transcription’ (GO:0006351).

**Fig. 6:**
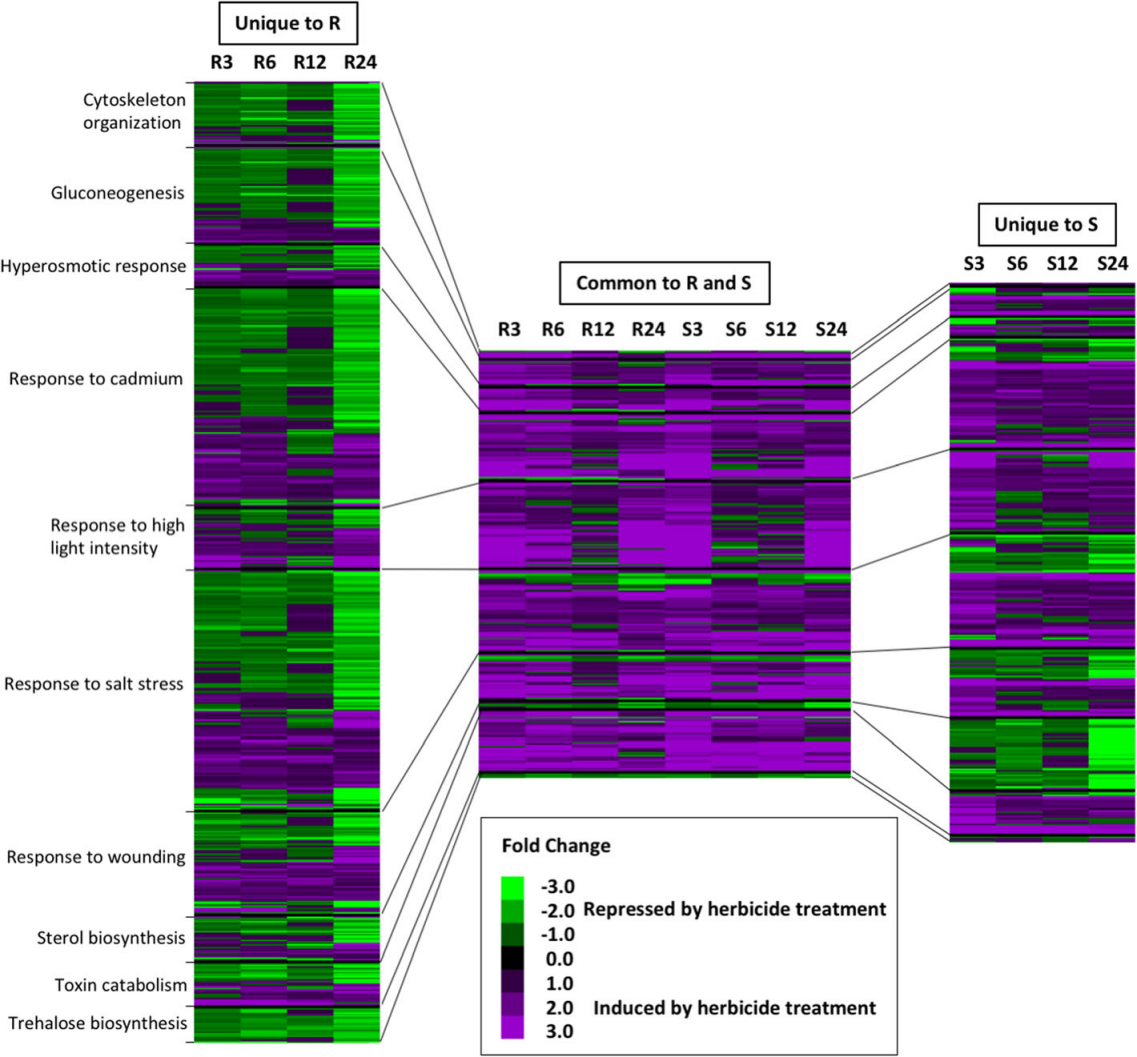
Characterizing DET expression patterns within a subset of significantly (FDR < 0.05, [43]) overrepresented GO terms. A core subset of ten gene ontology (GO) terms identified in Fig. 5 were chosen to examine the expression of differentially expressed transcripts (DETs) unique to the resistant (unique to R) or susceptible (unique to S) waterhemp genotypes or common to both (common to R and S). Black lines join portions of the heatmaps for a particular GO term of interest. While DETs common to both genotypes are largely induced, DETs unique to resistant or susceptible genotypes tend to have mixed expression patterns. Further, for some GO terms expression patterns are opposite between unique resistant and unique susceptible DETs. Overall, the expression pattern of induced versus repressed DETs is quite different across time

To understand how DETs in these GO terms responded to mesotrione treatment, we compared their expression patterns and expression profiles between resistant and susceptible waterhemp genotypes (Fig. 6). Of the 4799 total DETs, 1311 and 3034 were uniquely significantly differentially expressed in response to mesotrione treatment in the susceptible and resistant waterhemp genotypes, respectively. A total of 454 DETs were significantly differentially expressed in both genotypes. We compared DET expression patterns across ten overrepresented gene ontology terms identified above including ‘cytoskeleton organization’, ‘gluconeogenesis’, ‘hyperosmotic response’, ‘response to cadmium’, ‘response to high light intensity’, ‘response to salt stress’, ‘response to wounding’, ‘sterol biosynthesis’, ‘toxin catabolism’, and ‘trehalose biosynthesis’. When we examined the DETs common to both the resistant and susceptible waterhemp genotypes, we found that the majority of these genes were induced in both genotypes. However, in the susceptible genotype, expression was strongly induced 3 HAT, weakly expressed 6 and 12 HAT, and again strongly induced 24 HAT. A similar response occurred in the resistant genotype, however the dip in gene expression was largely restricted to the 12 HAT timepoint. In contrast, genes repressed in response to mesotrione were weakly repressed at 3, 6, and 12 HAT, but strongly repressed at 24 HAT.

For DETs unique to the susceptible or resistant waterhemp genotypes, we observed differences in the number and expression of DETs depending on the GO terms of interest. The GO terms ‘cytoskeleton organization’, ‘gluconeogenesis’, and ‘trehalose biosynthesis’ were largely unique to the resistant waterhemp genotype response and were repressed by mesotrione treatment. Aside from those DETs common to both genotypes, few DETs were observed in the susceptible waterhemp genotype. For the GO terms, ‘response to cadmium’, ‘response to salt stress’, ‘response to high light intensity’, and ‘toxin catabolism’, DETs unique to the susceptible waterhemp genotype were largely induced, while DETs associated with these GO terms in the resistant waterhemp genotype were largely repressed. Unique DETs associated with the GO term ‘sterol biosynthesis’ were repressed in the susceptible waterhemp genotype but had mixed expression in the resistant waterhemp genotype, while unique DETs associated with the GO terms ‘response to wounding’ and ‘hyperosmotic response’ had mixed expression among unique susceptible and unique resistant DETs.

### Clustering of herbicide-responsive DETs

**Fig. 7:**
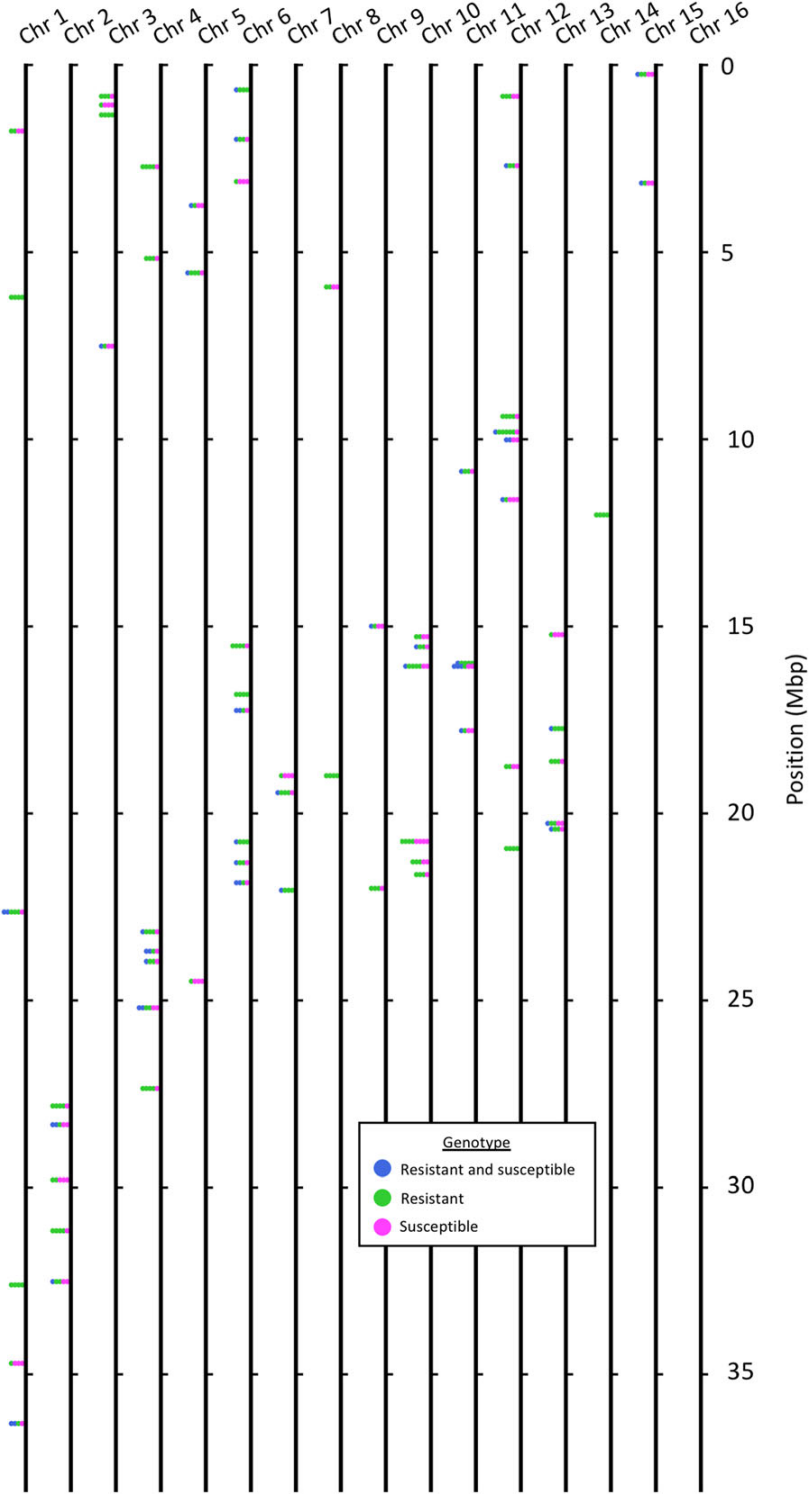
Clustering of waterhemp DETs based on predicted location relative to *Amaranthus hypochondriacus* genome. BLASTN (E < 10^− 20^, [27]) was used to predict relative transcript location based on ortholog location in the grain amaranth genome (Phytozome v12.1, *Amaranthus hypochondriacus* v2.1, [33]). Differentially expressed transcripts (DETs) were tested for overlap in a 100,000 bp window on center (± 50,000 bases) from the transcript start site. Neighboring DETs that fell into this window contributed to an average position of the cluster and the number of stacked transcripts. The DETs are color-coded based on the associated genotype(s). Blue circles indicate DETs associated with both the resistant and susceptible genotypes, green circles represent DETs associated with the resistant genotype and magenta circles represent DETs associated with the susceptible genotype. Transcript clusters were identified on all chromosomes except for chromosome 16

To identify DETs likely clustered in the waterhemp genome, we used reciprocal BLASTN (E < 10^− 20^, [27]) to predict transcript location relative to the grain amaranth genome (Phytozome v12.1, *Amaranthus hypochondriacus* v2.1, [33]) and then tested for overlap using a sliding window of 100,000 bp centered on transcription start sites. We identified 302 unique DETs that fell into 67 clusters (Fig. 7, Additional file 6). Sixty clusters had multiple genotypes, 7 clusters were unique to the resistant genotype, and no cluster was only associated with the susceptible genotype. DET clusters were identified on all grain amaranth chromosomes except for chromosome 16. The most clusters were found on grain amaranth chromosome 6 and the most unique DETs were found on grain amaranth chromosome 12. The biggest cluster of DETs consisted of 8 transcripts. Finding evidence of DET clustering relative to the grain amaranth genome suggests coordinate regulation of gene expression in response to herbicide treatment within clusters.

### Identification of candidate single nucleotide polymorphisms

**Fig. 8:**
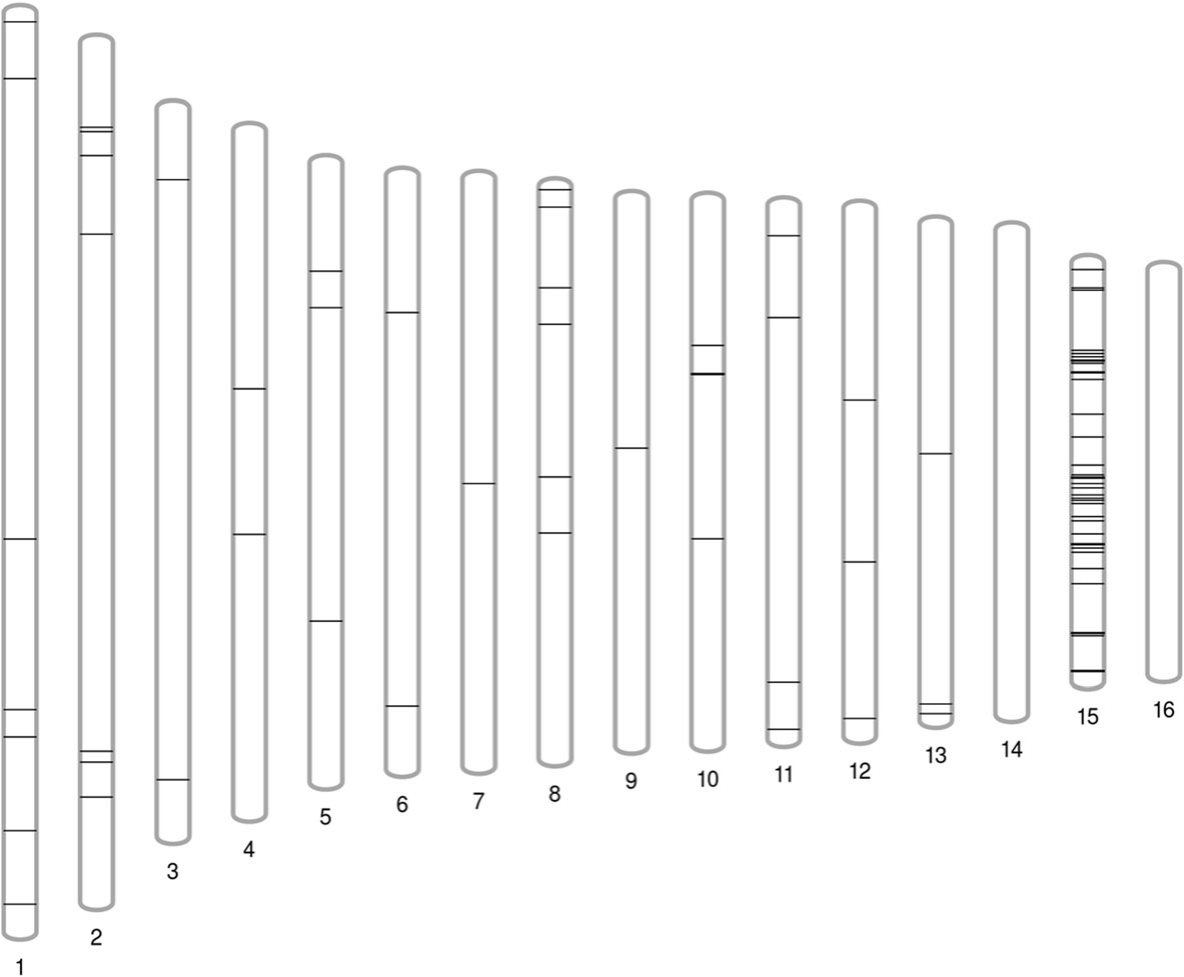
Mapping of candidate waterhemp single nucleotide polymorphisms (SNPs) relative to the *Amaranthus hypochondriacus* genome. SNPs were identified by calling SNPs between the resistant and susceptible genotypes relative to the waterhemp v4 transcriptome. BLASTN (E < 10^− 20^, [27]) was used to predict relative transcript and SNP location based on ortholog location in the grain amaranth genome (Phytozome v12.1, *Amaranthus hypochondriacus* v2.1, [33]). Of the 189 high-quality SNPs identified, 118 could be reliably mapped to the grain amaranth genome. The highest concentration of SNPs was found on chromosome 15

To identify candidate SNPs that could be used for mapping herbicide resistance and other traits in the future, we called variants between the resistant and susceptible genotypes relative to the v4 transcriptome. We identified 189 high-quality candidate SNPs (Fig. 8, Additional file 7). Accounting for multiple SNPs located on the same transcript we identified 137 transcripts that contained at least one SNP. To determine if SNP-containing transcripts were associated with specific functions, we queried the biological process GO terms associated with these transcripts. Identified GO terms included: ‘nuclear-transcribed mRNA catabolic process’ (GO:0000956), ‘DNA-templated regulation of transcription’ (GO:0006355), ‘protein glycosylation’ (GO:0006486), ‘response to xenobiotic stimulus’ (GO:0009410), ‘DNA-templated positive regulation of transcription’ (GO:0045893), ‘response to ethylene’ (GO:0009723), ‘response to abscisic acid’ (GO:0009737), ‘response to gibberellin’ (GO:0009739), ‘response to salicylic acid’ (GO:0009751), ‘response to jasmonic acid’ (GO:0009753), and ‘response to cadmium ion’ (GO:0046686). Collectively, these data suggest that SNP-containing transcripts can be associated with defense and stress responses.

To predict the location of SNP-containing transcripts we used reciprocal BLASTN (E < 10^− 20^, [27]) against transcripts from grain amaranth (Phytozome v12.1, *Amaranthus hypochondriacus* v2.1, [33]) to identify orthologous genes. SNPs for which a putative ortholog could not be confirmed were removed from the mapping analysis. We found candidate SNPs distributed across the grain amaranth genome (Fig. 8, Additional file 7). A high concentration of SNPs (64 of the 118 mapped) was located on chromosome 15. These SNPs correspond to 43 unique transcripts. Several of these transcript annotations were associated with defense responses. The SNP concentration and transcript annotations suggest this chromosome could be important for determining herbicide responses in resistant and susceptible populations.

## Discussion

Weeds are the major pest complex in global agricultural systems and will continue to be a problem given the increasing prevalence of herbicide resistance. Soybean and maize fields with high waterhemp population pressure can experience severe yield loss [2, 3]. Waterhemp is one of the most prolific and ecologically adapted herbicide resistant weeds found in agricultural fields of the Midwestern United States. Populations with evolved resistance to six different herbicide sites of action including acetolactate synthase (ALS, HG 2, EC 2.2.1.6) inhibitors, synthetic auxins (HG 4), photosystem II (PS II, HG 5, EC 1.10.3.9), 5-enolpyruvyl-shikimate-3-phosphate synthase (EPSPS synthase, HG 9, EC 2.5.1.19), protoporphyrinogen oxidase (PPO, HG 14, EC 1.3.3.4) and 4-hydroxyphenylpyruvate dioxygenase (HPPD, HG 27, EC 1.13.11.27) inhibiting herbicides have been identified [9]. Weediness characteristics associated with waterhemp include rapid growth, self-incompatibility, high seed output and dispersal and ability to compete for space and nutrients with crop species. These characteristics enhance the ability to evolve herbicide resistance and the potential for crop yield loss and subsequent economic impact on farmers and agricultural production. Thus, waterhemp is an important species to study by agricultural and plant science communities.

Several *Amaranthus* species, including waterhemp, have been discussed as potential candidates for genomic efforts [50, 51]. Supporting the waterhemp initiative was the identification of populations with five-way resistance to herbicides with different sites of action in Iowa in 2011 [52]. In this study, we used the mesotrione resistant population characterized by Kaundun et al. [15]. Mesotrione resistance in this population was not due to mutation, amplification, or increased expression of the herbicide target gene HPPD. Rather, biochemical analyses suggest cytochrome P450s contribute to increased herbicide metabolism. Significant differences in herbicide metabolism were not detected until 48 h after treatment. This suggests that herbicide responses are inducible and genes upstream of the cytochrome P450s directly or indirectly respond to herbicide treatment. To identify these early-acting genes and gene networks, we needed to establish genomic technologies for waterhemp. These tools are critically important for global food security [53].

### Development of genomic resources for studying traits of interest in waterhemp

Initial genomic studies of waterhemp using 454 pyrosequencing were able to characterize several herbicide resistance target genes [20, 21]. However, these studies pooled RNA samples of different tissues and treatments prior to sequencing, making it impossible to directly differentiate herbicide treatment responses in resistant and susceptible waterhemp genotypes. In contrast, our sequencing and de novo transcriptome assembly approach used 48 multiplexed libraries representing resistant and susceptible waterhemp genotypes, treated and mock-treated with the HPPD herbicide mesotrione across a twenty-four hour time course. Assembling RNA-seq data across libraries allowed us to develop a comprehensive waterhemp leaf transcriptome, which represents an important asset to various scientific communities. Comparisons of expressed genes in crop species verses weeds could provide insights into novel targets for weed specific genes for future weed control. Ecologists, plant and weed scientists could utilize this tool to study important weed traits or provide information on leaf development and photosynthesis that might be applied to crop improvement.

Assembly statistics of our waterhemp transcriptome (Table 1, Additional file 2) coupled with comparisons to predicted proteins from the model species *A. thaliana*, and the related species sugar beet and grain amaranth (Fig. 2) confirm the quality and breadth of our assembly. To increase the utility of the waterhemp transcriptome, the Additional data files include assembled sequences for the v4 assembly and a database of annotated transcripts. Furthermore, raw sequences have been deposited in the National Center for Biotechnology Small Reads Archive (NCBI SRA, Bioproject PRJNA432348 and SRA Study SRP132642) allowing for reassembly and continued improvement as more sequences from waterhemp populations become available.

To demonstrate how these data can be used by the weed science community, we mined the waterhemp transcriptome (Additional file 3) for any transcript containing ‘CYP’ or ‘P450’ in its annotation (Table 3). Recently, Kaundun et al. [15] attributed HPPD-resistance in the Tarnov, Nebraska population to increased herbicide metabolism through the activity of cytochrome P450s. We identified 970 putative cytochrome P450s. Similar mining of the DET file (Additional file 4) identified 79 herbicide responsive DETs with homology to cytochrome P450s. Of these 29 were significantly differentially expressed in the resistant population, 34 were significantly differentially expressed in the susceptible population and 16 were differentially expressed in both. Of great interest was transcript TR102135, which was the only transcript significantly induced in the resistant genotype at 3 hours. Ten cytochrome P450 transcripts were identified only in the resistant genotype at 24 HAT. Kaundun et al. [15], found the mesotrione metabolite 4-hydroxymesotrione began to accumulate in the resistant parent between 24 and 48 h after herbicide treatment. However, we detected differential expression of cytochrome P450s in the resistant population well before this time. This suggests genes upstream of cytochrome P450s can recognize and respond to herbicide treatment.

**Table 3:** Summary of candidate DETs for non-target site herbicide resistance and circadian rhythm associated proteins

	Keywords used in file search	Number of Hits	
		Whole Transcriptome	Differentially Expressed
Cytochrome P450	“CYP” “P450”	970	79
Glutathione Stransferase	“GST” “Glutathione Stransferase”	157	47
Glycosyltransferase	“glycosyltransferase” “glucosyltransferase”	689	69
ABC transporter protein	“ABC transporter” “ABC protein”	644	35
Circadian rhythm	“circadian”	833	42

The waterhemp transcriptome (Additional file 3) and significantly differentially expressed transcripts (Additional file 4) were mined for non-target site herbicide resistance and circadian rhythm associated transcripts using combinations of keywords to identify putative candidate transcripts for further investigation

Of the 79 herbicide-responsive transcripts with homology to cytochrome P450s, three were clustered relative to the grain amaranth genome (cluster 21, TR87604, TR34266 and TR3711, Additional file 6). We also identified two SNP-containing transcripts (TR33255 and TR9764) annotated as cytochrome P450s from our SNP data (Additional file 7). TR33255 was homologous to the *Arabidopsis* gene, AT2G29090.2, which encodes a cytochrome P450 (CYP707A2) with abscisic acid (ABA) 8′-hydroxylase activity [31, 54]. This protein belongs to the CYP707A gene family and is associated with controlling ABA levels in late seed maturation through germination [55]. ABA is a phytohormone involved in plant response to abiotic stress and has also been associated with protecting plants from herbicide damage [56, 57]. Devine et al. [57] demonstrated that applying exogenous ABA helped protect oats (*Avena sativa* L.) against applications of diclofop-methyl or low rates of tralkoxydim. While these transcripts did not respond to herbicide treatment in our analyses, they could represent non-inducible cytochrome P450s that could be differentially expressed between the resistant and susceptible genotypes or may be differentially expressed at timepoints not evaluated in this study. Similar approaches could be used to mine the data for sites-of-action for other important herbicides, non-target site herbicide resistance associated genes, or for traits of interest.

### Identification of transcripts responding to mesotrione treatment in waterhemp

Mapping of reads from specific waterhemp genotypes, mesotrione and mock treatments and time points allowed us to leverage our waterhemp transcriptome to identify the genes and gene networks responding to mesotrione treatment in waterhemp. A general trend we observed was that the number of DETs was greatest at 3 and 24 HAT (Fig. 3) in both the resistant and susceptible genotype, suggesting a biphasic response to herbicide treatment. In both the resistant and susceptible waterhemp genotypes, mesotrione treatment resulted in the differential expression of transcripts associated with stress and defense responses. When we compared gene expression patterns in genes common to the resistant and susceptible interaction (Fig. 6), we again see a biphasic response. Given that DETs in both waterhemp genotypes at 3 and 24 HAT were significantly overrepresented with GO terms associated with light (i.e., ‘response to high light intensity’, ‘response to red light’, and ‘response to far red light’), it’s possible that circadian rhythm could influence differential gene expression in response to herbicide treatment. However, the 3, 6 and 24 HAT timepoints were collected during daylight, so it seemed unlikely circadian rhythm was involved. To confirm this, we mined the DETs in each genotype by timepoint combination for the term ‘circadian’ (Table 3). We identified 28 and 17 herbicide responsive DETs with annotations associated with circadian rhythm in the resistant and susceptible genotype, respectively. In the resistant genotype, 0, 0, 0, and 15 circadian clock associated DETs were expressed specifically at R3, R6, R12, and R24. In the susceptible genotype, we identified 8, 1, 1, and 5 circadian clock associated DETs at S3, S6, S12, and S24, respectively. This suggests herbicide treatment could impact circadian rhythm.

Biphasic gene expression patterns have been observed in response to biotic (reviewed by [58]) and abiotic [59, 60] stress and in response to mechanical wounding [61]. Reactive oxygen species (ROS) have now been tied to biphasic gene expression in defense, response to wounding, high light conditions, and abiotic stress (reviewed by [62]). HPPD-inhibitor herbicides disrupt photosynthesis and the production of carotenoids that protect plants from UV damage leading to the accumulation of ROS [63]. The GO terms ‘response to hydrogen peroxide’ (FDR < 0.05, R, R3, R24, S, S3, and S24) and ‘response to reactive oxygen species’ (FDR < 0.05, R3, R, and S) were both significantly overrepresented in resistant and susceptible genotypes, suggesting that herbicide treatment can also induce biphasic responses in weeds, likely through the action of ROS.

When we compared DET expression in transcripts unique to the resistant or susceptible waterhemp genotypes, we observed that DETs associated with ‘response to cadmium’, ‘response to high light’, ‘hyperosmotic response’, ‘response to salt stress’, and ‘toxin catabolism’ were largely induced by mesotrione treatment in the susceptible genotype across the time course. In contrast, DETs unique to the resistant waterhemp genotype and associated with these same GO terms were largely repressed across time (Fig. 6). These data highlight several remarkable features of the resistant waterhemp genotype’s response to mesotrione. First, responses to mesotrione treatment were detected very quickly, within three HAT in both resistant and susceptible waterhemp genotypes. Second, while the susceptible waterhemp genotype continued to induce stress responses over the experiment time course, by three HAT the resistant waterhemp genotype was already repressing differential expression of stress-associated genes. This suggested that by three HAT, the resistant waterhemp genotype began to neutralize herbicidal activity and was likely returning to normal physiological function.

Using the reference genome of grain amaranth, we were able to identify groups of DETs that clustered together by predicted genomic locations. The clustered DETs were generally associated with detoxification and stress responses. Toxin metabolism in plants occurs in three phases: transformation, conjugation and compartmentation. The two major enzymes associated with phase I and phase II of detoxification (cytochrome P450 monooxygenase and glutathione transferase, respectively) were among the clustered DETs [64]. Among the annotations for the clustered DETs we found multiple enzymes associated with all three phases of pesticide and xenobiotic metabolism such as alcohol or aldehyde dehydrogenase, glycosyltransferase, methyltransferase, and ATP-binding cassette (ABC) transporters [65] (Table 3). Other DETs have associations with stress response and signaling such as heat shock proteins and lipoxygenase [66, 67]. These associations of clustered DETs with xenobiotic detoxification and stress signaling support potential coordinate regulation of defense response genes by waterhemp in response to mesotrione treatment.

We cross-referenced the 189 SNP-containing transcripts with the total 4799 herbicide responsive DETs. We identified 18 unique herbicide responsive transcripts that contained SNPs. Four transcripts with documented herbicide response were easily identifiable. TR45906|c2_g1_i2 was homologous to the *Arabidopsis* gene CYP73A5, a member of the cytochrome P450 enzyme family [68]. TR16523|c2_g1_i4 and TR36394|c2_g7_i1 were homologous to the UDP-glucosyl transferase, UGT73B3. Lim et al. [69] found UGT73B3 recessive mutants resistant to paraquat, a photosystem I (PSI, HG 22, EC 1.97.1.12) electron diverting herbicide. TR18103|c1_g2_i1 was homologous to UGT74E2, which was also reported in the differential response of *Arabidopsis* to ALS-inhibiting (HG 2) and EPSPS-inhibiting (HG 9) herbicides [70]. Given that these SNPs correspond to herbicide responsive DETs, they are high priority candidates for future marker development.

**Fig. 9:**
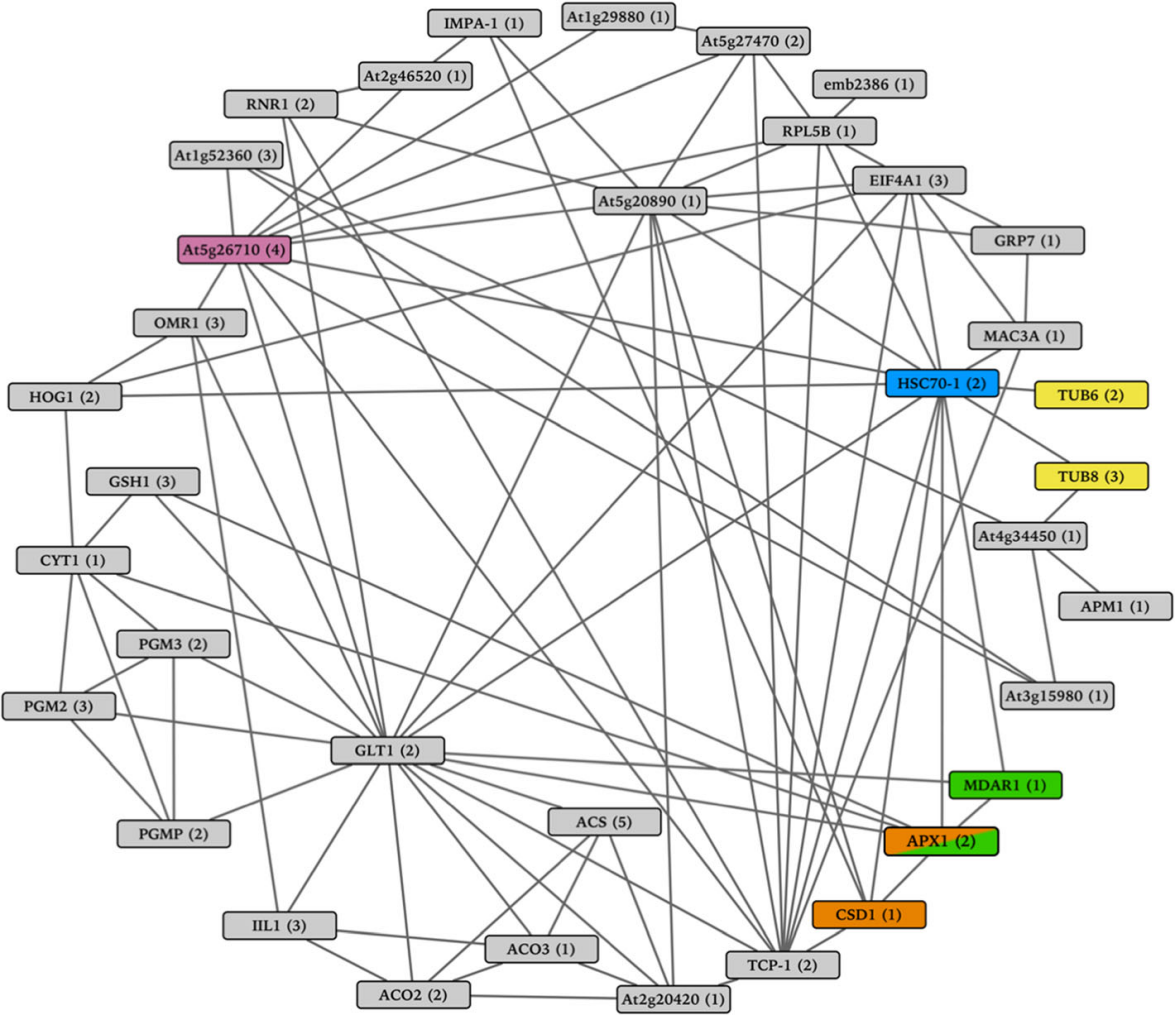
Identification of gene networks unique to mesotrione resistance associated with the GO terms gluconeogenesis, cytoskeleton organization and trehalose biosynthesis. We took advantage of the waterhemp (*Amaranthus tuberculatus*) annotation platform to identify the best *Arabidopsis thaliana* homologs for 91 unique resistant DETs associated with these GO terms. Forty-five unique *A. thaliana* identifiers were then submitted to the STRING website to identify potential gene networks [71]. All 45 unique *A. thaliana* identifiers were present in the database and 36 could be combined in a single network. Lines linking proteins indicate connections between proteins. The numbers in parentheses indicate the number of unique resistant DETs that correspond to each *Arabidopsis* gene ID. The yellow nodes highlight genes that have associations with dinitroaniline (HG 3) herbicide resistance, the purple node highlights an association with 2,4-D (HG 4) herbicide resistance, the orange nodes highlight associations with atrazine (HG 5) herbicide resistance and the green nodes highlight associations with paraquat (HG 22) herbicide resistance. The blue node highlights the only node in this network that interacted with all six nodes associated with herbicide resistance

While responses to light and stress were expected, our analyses determined that DETs unique to the resistant waterhemp genotype were significantly overrepresented with the GO terms ‘cytoskeleton organization’, ‘gluconeogenesis’, and ‘trehalose biosynthesis’. Genes within these GO terms were significantly repressed in response to the mesotrione treatment, especially at 24 HAT. To connect these responses and examine the underlying gene networks, we took advantage of the waterhemp annotation platform to identify the best *A. thaliana* homologs for DETs associated with these GO terms. Unique *A. thaliana* identifiers were then submitted to the STRING website to identify gene networks [71]. Networks in STRING are established using a variety of methods including but not limited to experiments, public databases and co-expression. Collectively, the three GO terms contained 91 DETs which corresponded to 45 unique *A. thaliana* identifiers. Of these, 36 could be assigned to the same network with a confidence score ranging from 0.41 to 0.99 (Fig. 9). Interestingly, the network also contained several genes associated with herbicide resistance. This included two beta-tubulins inhibitors (*AtTUB6* and *AtTUB8*), a glutamyl-tRNA synthetase (*At5g26710*), an ascorbate peroxidase (*APX1*), a superoxide dismutase (*CSD1*) and a monodehydroascorbate reductase (*MDAR1*). Anthony et al. [72] found that co-overexpression of mutant alpha and beta tubulins generated dinitroaniline (HG 3) herbicide-resistant tobacco (*Nicotiana tabacum* L.). While studying gene expression patterns of naturally tolerant wheat (*Triticum aestivum* L., variety Greina), Pasquer et al. [73] found glutamyl-tRNA synthetase was significantly induced by 2,4-D (2,4-dichlorophenoxyacetic acid, HG 4). Jiang et al. [74] found increased expression of superoxide dismutase and ascorbate peroxidase in response to atrazine (HG 5) treatment in pearl millet (*Pennisetum americanum* (L.) K. Schum). Murgia et al. [75] found that overexpression of ascorbate peroxidase in *Arabidopsis* also conferred resistance to paraquat (HG 22). Monodehydroascorbate reductase activity was correlated to paraquat (HG 22) resistance in horseweed (*Conyza canadensis* (L.) Cronquist) [76]. Interestingly, all six gene nodes interacted with the heat shock 70 kDa protein (*HSC70–1*) node in our network (Fig. 9). Additionally, two genes were identified that had associations with herbicide resistance but did not interact with the larger gene network, a trehalose-6-phosphate phosphatase (*TPPD*) and a regulatory particle AAA-ATPase 2A (*RPT2a*) [77, 78]. These findings, mirroring the expression we observe in the waterhemp DETs, suggest that many herbicides thought to have unique targets may actually be acting on the same gene networks.

## Conclusions

Many important plant species to agriculture lack well-curated reference genomes. However, RNA-seq has allowed scientific communities to develop transcriptomes for characterizing genes and traits important for agronomic performance of both crops and weeds. In this study, we provide a comprehensive resource for waterhemp genomics, including the first waterhemp transcriptome, identification of DETs responding to herbicide treatment in HPPD-resistant and susceptible genotypes and candidate SNPs for future marker development. While other studies have sequenced the waterhemp transcriptome and identified important herbicide target-sites, this is the first transcriptomic analysis that identifies genes and gene networks that are differentially expressed in response to HPPD inhibiting herbicides in HPPD-resistant and susceptible waterhemp. Our analyses reveal 1) that waterhemp responses to mesotrione are quick and detectable in as little as 3 hours, 2) differential gene expression in resistant and susceptible waterhemp genotypes show little overlap in mesotrione responses, 3) genes targeted by other herbicides may belong to the same gene networks providing insight into the evolution of herbicide resistance and 4) predicted locations of DETs suggest coordinate regulation of defense responses in HPPD-resistant waterhemp. These findings lay a strong foundation for future research and will improve opportunities to better understand and manage weeds with evolved resistances to herbicides.

## Additional files

Additional file 1: Contig length distribution of the four versions of the de novo waterhemp (*Amaranthus tuberculatus*) transcriptome assemblies. Assemblies v1, v2 and v3 were generated with differing kmer lengths (25, 29, and 32, respectively) as described in the materials and methods (PNG 175 kb)

Additional file 2: Waterhemp (*Amaranthus tuberculatus*) v4 transcriptome sequences in FASTA format. (XLSX 21386 kb)

Additional file 3: Annotation of the waterhemp (*Amaranthus tuberculatus*) version 4 transcriptome. BLASTX [26] was used to compare waterhemp transcriptome sequences against the Uniref100 (version 01/22/2016, [31]) nonredundant protein database. (XLSX 919 kb)

Additional file 4: dentification of waterhemp (*Amaranthus tuberculatus*) transcripts significantly (FDR < 0.05, [43]) differentially expressed in response to mesotrione treatment. edgeR was used to identify differentially expressed transcripts (DETs) responding to herbicide treatment relative to mock-treated controls within each genotype (herbicide resistant (R) and susceptible(S)) and at specific time points (3, 6, 12, and 24 h after treatment (HAT). (XLSX 18 kb)

Additional file 5: Characterization of waterhemp (*Amaranthus tuberculatus*) differentially expressed transcripts (DETs) using gene ontology overrepresentation. A Fisher’s Exact Test [42] with a Bonferroni correction [43] was used to identify significantly (*P* < 0.05) overrepresented gene ontology biological process terms among DETs significant within a genotype at a particular time or across time, relative to all transcripts in the waterhemp v4 transcriptome. (XLSX 141 kb)

Additional file 6: Waterhemp (*Amaranthus tuberculatus*) differentially expressed transcripts (DETs) cluster relative to *Amaranthus hypochondriacus* genome. BLASTN (E < 10^− 20^, [26]) was used to predict relative transcript location based on ortholog location in the grain amaranth genome (Phytozome v12.1, *Amaranthus hypochondriacus* v2.1, (XLSX 73 kb)

Additional file 7: Mapped candidate single nucleotide polymorphisms (SNPs) identified between mesotrione-resistant and susceptible waterhemp (*Amaranthus tuberculatus (GZ 19875 kb)*

## Abbreviations

ABA: abscisic acid
ABC: ATP-binding cassette
ACC: aminocyclopropane-1-carboxylic acid
ALS: aceltolactate synthase
COC: crop oil concentrate
contig: contiguous overlapping sequences
CPM: count per million
CYP: cytochrome P450
DETs: differentially expressed transcripts
EPSPS: 5-enolpyruvyl-shikimate-3-phosphate
FDR: false discovery rate
GFF: general feature format
GO: gene ontology
GOBP: gene ontology biological process
HAT: hours after treatment
HG: herbicide group
HGA: 2,5-dihydroxyphenylacetate
HPP: 4-hydroxyphenylpyruvate
HPPD: 4-hydroxyphenylpyruvate-dioxygenase
log_2_FC: log_2_ fold change
NCBI SRA: National Center for Biotechnology Small Reads Archive
ORF: open reading frame
PPO: protoporphyrinogen oxidase
PS: photosystem
RNA-seq: RNA-sequencing
ROS: reactive oxygen species
RSEM: RNA-seq by expectation-maximization;
SNP: single nucleotide polymorphism
TAIR10: The Arabidopsis Information Resource version 10
TMM: trimmed mean of M-values
UAN: urea ammonium nitrate
