# Supplementary figures and images for: Using RNA-seq to characterize responses to 4-hydroxyphenylpyruvate dioxygenase (HPPD) inhibitor herbicide resistance in waterhemp (Amaranthus tuberculatus)
Source: BMC Plant Biol. 2019 May 6;19:182. doi: 10.1186/s12870-019-1795-x (PMC6501407; doi:10.1186/s12870-019-1795-x)

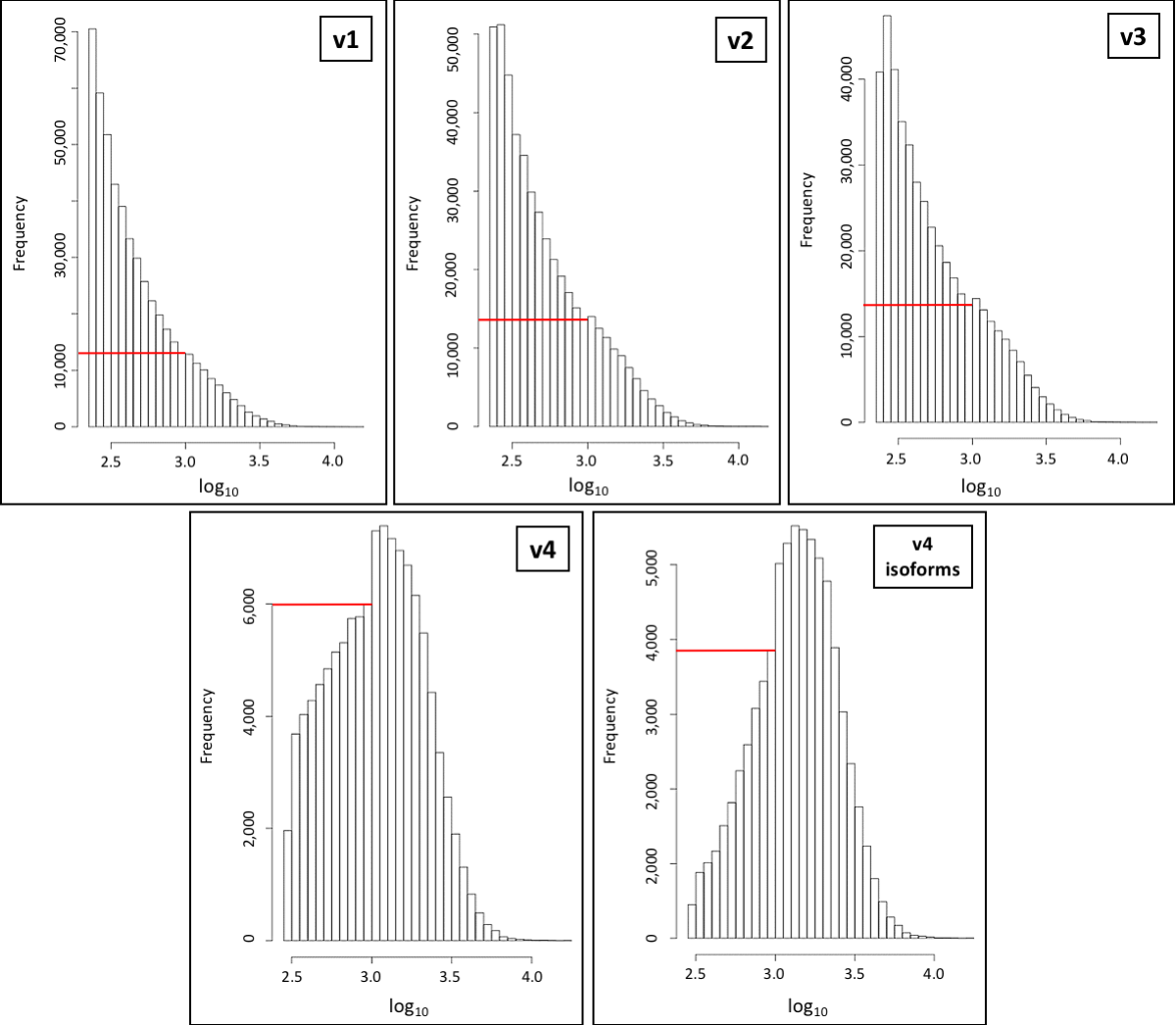

Supplement: Supplementary file 1 — Contig length distribution of the four versions of the de novo waterhemp (Amaranthus tuberculatus) transcriptome assemblies. Assemblies v1, v2 and v3 were generated with differing kmer lengths (25, 29, and 32, respectively) as described in the materials and methods (PNG 175 kb) [file 12870_2019_1795_MOESM1_ESM.png]
